# MicroRNA-552 Accelerates the Progression of Gastric Cancer by Targeting FOXO1 and Regulating PI3K/AKT Pathway

**DOI:** 10.1155/2021/9966744

**Published:** 2021-05-04

**Authors:** Yuguo Zhao, Jianwen Zhang, Wenbin Yang, Zhao Yang, Kaikai Zhou

**Affiliations:** Department of Gastrointestinal Surgery, The First People's Hospital of Chenzhou, Chenzhou, Hunan Province 423000, China

## Abstract

The specific function of microRNA-552 (miR-552) has been investigated in several malignancies, except gastric cancer (GC). Therefore, this study was performed to determine the role of miR-552 in GC.GC tissues and adjacent non-tumor tissues were collected to determine the expressions of miR-552. Quantitative real-time polymerase chain reaction assays (RT-qPCR) and Western blot analysis were carried out to measure expression levels. The regulatory mechanism of miR-552 was explored by (3-(4,5-dimethylthiazol-2-yl)-2,5-diphenyl tetrazolium bromide) MTT Assay, and Transwell assays. The binding site between miR-552 and FOXO1 was verified by dual-luciferase reporter assays. Upregulation of miR-552 expression was detected and associated with worse clinical outcomes in GC. Furthermore, high miR-552 expression predicted poor prognosis in GC patients. Functionally, upregulation of miR-552 promoted cell viability, metastasis, epithelial-mesenchymal transition (EMT), and phosphatidylinositol 3-kinase and protein kinase B (PI3K/AKT) pathway in GC. In addition, miR-552 was confirmed to target forkhead box O1 (FOXO1) directly and inversely regulate its expression in GC. Upregulation of FOXO1 reversed the carcinogenesis of miR-552 in GC. In conclusion, miR-552 serves as a tumor promoter in GC through targeting FOXO1 and regulating EMT and PI3K/AKT pathway.

## 1. Introduction

Gastric cancer (GC) is the second largest cancer, second only to lung cancer. Most patients with GC are over 50 years old, and men are twice as likely to have GC as women [1]. GC is more common in Japan and China, mainly due to dietary reasons. Moreover, the development of GC is extremely fast, and the current treatment can only control the spread of GC [2]. The therapeutic effect of GC is related to the onset, the pathological type, the thoroughness of surgical radicalization, and the comprehensive treatments [3]. Early GC has the best therapeutic effect, but there is a risk of recurrence. Nearly two-thirds of recurrence will lead to distant metastasis of GC, and distant metastasis is the biggest cause of death in GC patients [4]. Therefore, it is of great significance to explore potential molecular markers for the early diagnosis and treatment of GC.

MicroRNAs (miRNAs) are well-known to be involved in human diseases and cancers [5]. Moreover, many miRNAs have been reported to regulate biological activities in GC. For example, miR-423-5p was upregulated in GC and promoted cancer growth and metastasis [6]. An et al. proposed that miR-1236-3p was downregulated in GC and inhibited invasion and metastasis [7]. Now, the dysregulation of miR-552 caught our attention, which has not been investigated in GC. Miao et al. reported that miR-552 suppressed both transcription and translation of cytochrome P450 2E1 [8]. In addition, miR-552 can distinguish primary lung adenocarcinoma and colorectal cancer metastases [9]. MiR-552 was upregulated in osteosarcoma and hepatocellular carcinoma and promoted cell viability and metastasis [10, 11]. Besides that, increased expression of miR-552 was found to act as a potential predictor biomarker for poor prognosis of colorectal cancer [12]. Previous studies indicate that miR-552 participates in the pathogenesis of human cancers.

As a forkhead box transcription factor, forkhead box O1 (FOXO1) has been found to participate in cancer development [13]. For example, the expression of FOXO1 predicted disease-free survival in breast cancer [14]. Xie et al. found that FOXO1 was a tumor suppressor in classical Hodgkin lymphoma [15]. Moreover, FOXO1 controlled thyroid cell proliferation and was involved in thyroid tumorigenesis [16, 17]. In the meantime, the interaction between FOXO1 and miRNAs has been detected in some malignancies, such as bladder cancer and breast cancers [17, 18]. FOXO1 has been proposed to play an essential role in PI3K/AKT signaling and regulate many biological activities in cancers [19]. It was reported that miR-132 played an oncogenic role in laryngeal squamous cell carcinoma by targeting FOXO1 and activating the PI3K/AKT pathway [20]. However, the regulatory mechanism of miR-552/FOXO1/PI3K/AKT remains unclear in GC. Therefore, the dysregulation of miR-552 and its regulatory mechanism in GC were evaluated in this study. These findings could provide new insights into GC treatment.

## 2. Materials and Methods

### 2.1. Sample Collection

In this study, GC specimens and normal specimens were obtained from 84 patients at The First People's Hospital of Chenzhou, Chenzhou, Hunan, China. Before the experiment, written informed consent was provided by all GC patients. The participants did not receive any treatment except for surgery. This study was approved by the Institutional Ethics Committee of The First People's Hospital of Chenzhou.

### 2.2. Cell Lines and Transfection

Normal gastric cell GES-1 and MKN-45, MGC-803 GC cell lines (BNCC, Beijing, China) were seeded in RPMI-1640 medium containing 10% fetal bovine serum (FBS). These cells were cultured at 37°C in 5% CO_2_.

MiR-552 mimics or inhibitor, FOXO1 vector (RiboBio, Guangzhou, China) were severally transferred into MKN-45 cells using Lipofectamine 2000 (Invitrogen, Carlsbad, USA). Untreated MKN-45 cells were set as the control.

### 2.3. RT-qPCR

The extraction of total RNA was performed using TRIzol reagent (Invitrogen, Carlsbad, USA). The cDNA was synthesized by PrimeScript RT reagent (Takara, Dalian, China). We conducted RT-qPCR using SYBR Green Master Mix II (Takara) based on the manufacturer's instructions. MiR-552 or FOXO1 was normalized by U6 or GAPDH as the internal reference. Their expression levels were calculated using the 2^−△△*ct*^ method. The primers used in our work were as follows: miR-552, forward primer: 5′-GTT TAA CCT TTT GCC TGT TGG-3′, reverse primer: 5′-CGA ACG CTT CAC GAA TTT G-3'; U6, forward primer: 5′-CTC GCT TCG GCA GCA CA-3′, reverse primer: 5′-AAC GCT TCA CGA ATT TGC GT-3'; FOXO1 forward primer: 5′-AGG GTT AGT GAG CAG GTT ACA C-3′, reverse primer: 5′-TGC TGC CAA GTC TGA CGA AA-3'; GAPDH forward, 5′-ACA TCG CTC AGA CAC CAT G-3′, reverse, 5′-TGT AGT TGA GGT CAA TGA AGG G-3'.

### 2.4. MTT Assay

Transfected MKN-45 cells (4 × 10^3^ cells/well) were seeded in RPMI-1640 with 10% FBS for 24, 48, 72 or 96 h. Next, the suspension of MKN-45 cells was added with 20 *μ*l of MTT for 4 h. Then, 150 *μ*l of dimethyl sulfoxide was added to the medium. After 10 minutes, cell viability was assessed using a microplate reader (Olympus Corp., Tokyo, Japan) to determine the optical density at 490 nm.

### 2.5. Transwell Assay

Transwell assay was used to assess cell migration and invasion abilities. Next, upper chamber was added with Matrigel (BD Biosciences, Franklin Lakes, NJ, USA) to detect MKN-45 cell invasion. The transfected cells (5 × 10^3^ cells/well) were put in the upper chamber, and lower chamber filled with 10% FBS. The migrated or invaded cells were fixed with methanol and stained with 0.1% crystal violet for 30 mins. Finally, migrated or invaded cells were examined under a light microscope (Olympus Corporation, Tokyo, Japan). Cell migration assay was performed without Matrigel, and other process was the same as cell invasion assay.

### 2.6. Western Blot Analysis

First, the protein sample was lysed using RIPA buffer (Beyotime, Shanghai, China). Then, the supernatant was collected as the total protein. The protein was electrophoresed by 10% SDS-PAGE. The protein was blocked by 5% non-fat milk for 1 h. After incubating the protein with the following primary antibodies (Bax, Bcl-2, E-cadherin, N-cadherin, PI3K, AKT and GAPDH) overnight at 4°C, the diluted secondary antibodies were added to incubate protein for another 1 h. Finally, the protein was examined by an ECL reagent (Millipore, MA, USA).

### 2.7. Luciferase Reporter Assay

Dual-luciferase reporter assay (Promega, Madison, WI, USA) was performed to verify the relationships between miR-552 and FOXO1.The 3′-UTR of wild or mutant FOXO1 was inserted into pcDNA3.1 plasmid vector (Promega, Madison, USA) to construct the luciferase reporter vectors of Wt-FOXO1 and Mut-FOXO1. The above vectors were then severally transfected into MKN-45 cells with miR-552 mimics or NC-mimics using Lipofectamine 2000 (Invitrogen) to execute the dual-luciferase reporter assay. After incubation of 48 h, a dual-luciferase assay system (Promega, USA) was used to detect luciferase activities.

### 2.8. Statistical Analysis

Data are shown as mean ± SD, which were analyzed using SPSS 17.0 or Graphpad Prism 6. Chi-squared test, one-way analysis of variance with Tukey's post hoc test, and univariate Kaplan–Meier method with the log-rank test were applied to calculate differences between groups. Differences were considered as significant at *p* < 0.05.

## 3. Results

### 3.1. The Expression of miR-552 Was Increased in GC Tissues

**Figure 1:**
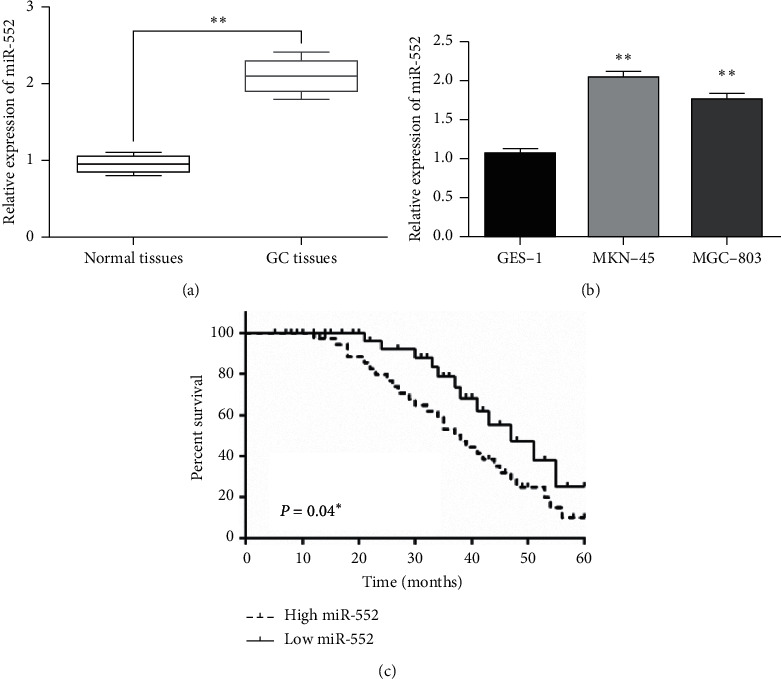
The expression of miR-552 was increased in GC tissues. (a) MiR-552 expressions in GC tissues. (b) The miR-552 expression in MKN-45 and MGC-803 cells compared to that in GES-1 cells. (c) High miR-552 expression was associated with poor prognosis in GC patients. ^*∗∗*^*p* < 0.01.

The alternation of miR-552 expression was initially detected in GC tissues. RT-qPCR showed that miR-552 expression was increased in GC tissues compared to normal tissues (*p* < 0.01, Figure 1(a)). Similarly, miR-552 was higher in MKN-45 and MGC-803 cells than in GES-1 cells (*p* < 0.01, Figure 1(b)). MKN-45 cells were selected for the functional assay due to the significant difference in miR-552 expression. In addition, we analyzed the correlation between abnormal miR-552 expression and clinical features in GC patients. GC patients were assigned into high and low miR-552 groups based on the median miR-552 expression level. As shown in Table 1, the dysregulation of miR-552 was associated with differentiation (*p* < 0.01), TNM stage (*p* < 0.05), and lymph node metastasis (*p* < 0.05). Furthermore, GC patients with high miR-552 expression showed a shorter overall survival, indicating that upregulating of miR-552 predicted poor prognosis in GC patients (*p* < 0.01, Figure 1(c)). These results indicated that miR-552 might function as an important regulator in the pathogenesis of GC.

### 3.2. Upregulation of miR-552 Promoted Cell Viability and Metastasis in GC

**Figure 2:**
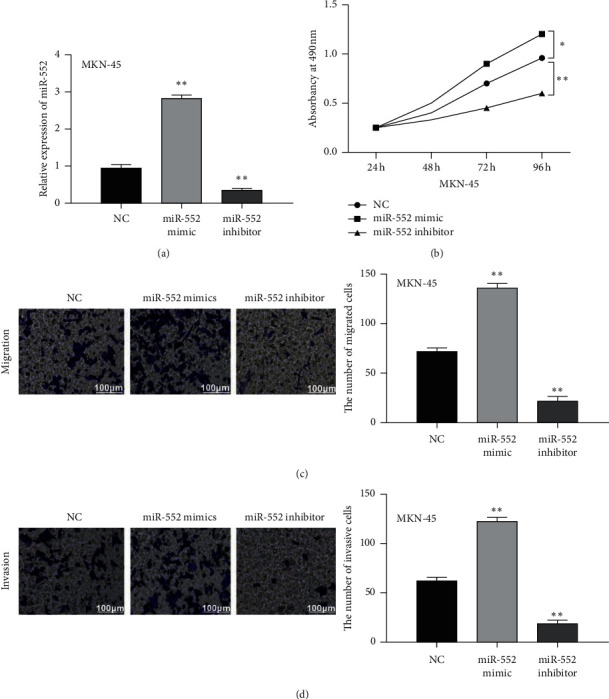
Upregulation of miR-552 promoted cell viability and metastasis in GC. (a) MiR-552 expression in MKN-45 cells with its mimics or inhibitor. (b–d) Cell proliferation, migration, and invasion regulated by miR-552 mimics or inhibitor. ^*∗*^*p* < 0.05, ^*∗∗*^*p* < 0.01.

Next, miR-552 mimics or inhibitor was transfected into MKN-45 cells to perform a gain-loss experiment. MiR-552 expression was promoted by its mimics and inhibited by its inhibitor in MKN-45 cells (*p* < 0.01, Figure 2(a)). MTT assay revealed that overexpression of miR-552 promoted cell proliferation, whereas downregulation of miR-552 restrained MKN-45 cell proliferation (*p* < 0.05, Figure 2(b)). Transwell assay displayed that cell migration was accelerated by miR-552 mimics and repressed by miR-552 inhibitor in MKN-45 cells (*p* < 0.01, Figure 2(c)). Similarly, upregulation of miR-552 facilitated cell invasion, while downregulation of miR-552 inhibited cell invasion in MKN-45 cells (*p* < 0.01, Figure 2(d)). Taken together, miR-552 promoted the viability and metastasis of GC cells.

### 3.3. MiR-552 Activated EMT and PI3K/AKT Pathway in GC

**Figure 3:**
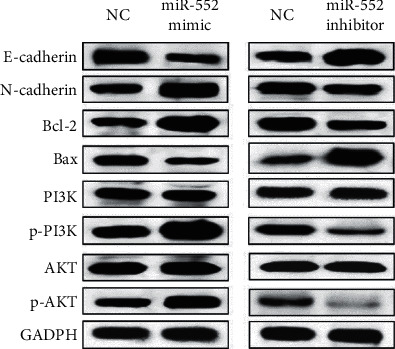
MiR-552 activated EMT and PI3K/AKT pathway in GC. MiR-552 regulated expressions of E-cadherin, N-cadherin, Bax, Bcl-2, PI3K, and AKT in MKN-45 cells.

In addition, the effect of miR-552 on the EMT and PI3K/AKT pathway was investigated to further illuminate its role in GC. As for EMT, miR-552 mimics were found to promote N-cadherin expression and inhibit the expression of E-cadherin. However, miR-552 inhibitor reduced N-cadherin expression and facilitated E-cadherin expression in MKN-45 cells (Figure 3). Next, expressions of apoptosis-associated proteins (Bcl-2/Bax) were measured in MKN-45 cells with miR-552 mimics or inhibitor. The results showed that miR-552 mimics declined Bax expression and promoted survival gene Bcl-2 expression. Furthermore, miR-552 inhibitor promoted Bax expression and reduced Bcl-2 expression (Figure 3). Besides that, expressions of p-PI3K and p-AKT were found to be promoted by the upregulation of miR-552 and suppressed by the downregulation of miR-552. However, expressions of PI3K and AKT were not affected by miR-552 in MKN-45 cells (Figure 3). Combining all these results, miR-552 was considered to serve as a cancerogenic factor in GC progression.

### 3.4. FOXO1 Is a Direct Target of miR-552

**Figure 4:**
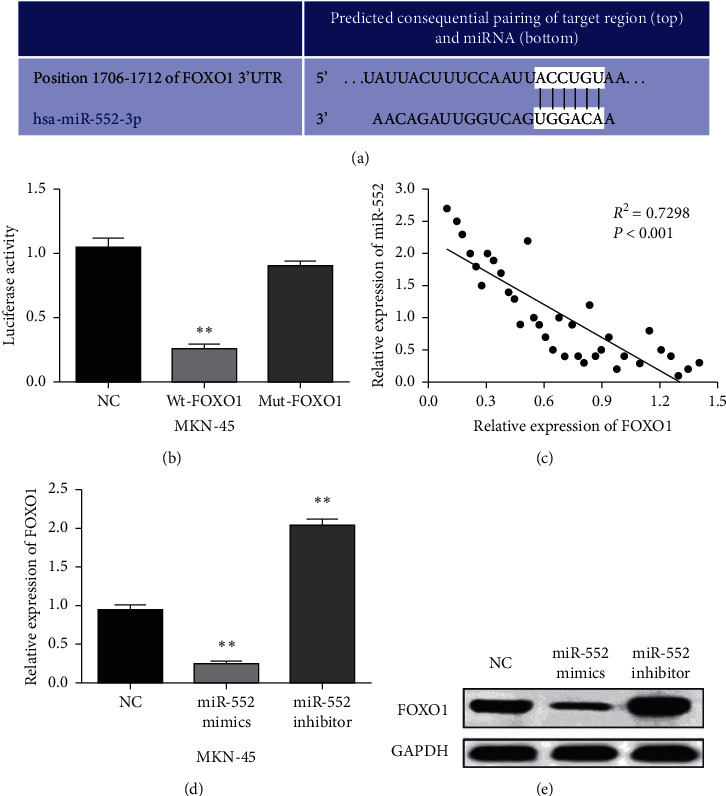
FOXO1 is a direct target of miR-552. (a) The binding sites between miR-552 and FOXO1. (b) Luciferase reporter assay. (c) A negative correlation between miR-552 and FOXO1. (d, e) FOXO1 expression in MKN-45 cells with miR-552 mimics or inhibitor. ^*∗∗*^*p* < 0.01.

The downstream target of miR-552 was searched in TargetScan (http://www.targetscan.org/) databases to explain its regulatory mechanism in GC. It predicts that miR-552 has binding sites with the 3′-UTR of FOXO1 (Figure 4(a)). Luciferase reporter assay was designed to confirm this prediction. We found that miR-552 mimics decreased Wt-FOXO1 luciferase activity but had no effect on Mut-FOXO1 luciferase activity in MKN-45 cells (*p* < 0.01, Figure 4(b)). Furthermore, miR-552 has a negative correlation with FOXO1 expression in GC tissues (*p* < 0.0001, *R*^2^ = 0.7298; Figure 4(c)). In addition, the expression level of FOXO1 was reduced by upregulation of miR-552 and enhanced by downregulation of miR-552 in MKN-45 cells (*p* < 0.01, Figures 4(d) and 4(e)). Briefly, miR-552 directly targets FOXO1 and has negative association with FOXO1 expression in GC.

### 3.5. Upregulation of FOXO1 Reversed the Carcinogenesis of miR-552 in GC

**Figure 5:**
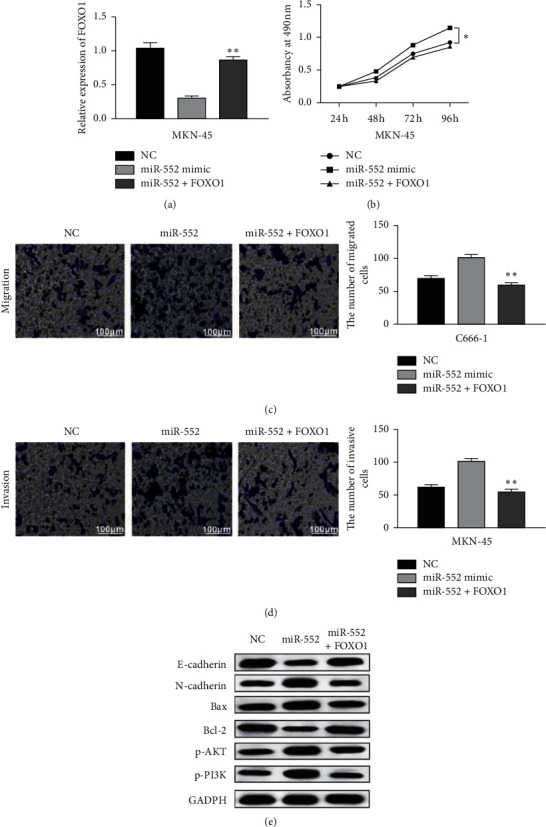
Upregulation of FOXO1 reversed the carcinogenesis of miR-552 in GC. (a) FOXO1 expression in MKN-45 cells with miR-552 mimics and FOXO1 vector. (b–d) Cell proliferation, migration, and invasion in MKN-45 cells with miR-552 mimics and FOXO1 vector. (e) Expressions of E-cadherin, N-cadherin, Bax, Bcl-2, PI3K, and AKT in MKN-45 cells with miR-552 mimics and FOXO1 vector. ^*∗*^*p* < 0.05, ^*∗∗*^*p* < 0.01.

Finally, the interaction between miR-552 and FOXO1 was investigated in MKN-45 cells with miR-552 mimics and FOXO1 vector. RT-qPCR displayed that the FOXO1 vector restored its decreased expression induced by miR-552 mimics (Figure 5(a)). Functionally, upregulation of FOXO1 impaired the promoted effect of miR-552 on cell proliferation (Figure 5(b)). Similarly, the promoted effect of miR-552 on cell migration and invasion was also abolished by the FOXO1 vector (Figures 5(c) and 5(d)). In addition, the reverse effect of FOXO1 on EMT and PI3K/AKT pathway was also identified in MKN-45 cells (Figure 5(e)). Collectively, upregulation of FOXO1 weakened the carcinogenesis of miR-552 in GC.

## 4. Discussion

Recently, various miRNAs have been found to participate in tumorigenesis of GC. For example, miR-208a was upregulated and acted as a tumor promoter in GC [21]. In the current study, the expression of miR-552 was increased in GC tissues, which was related to worse clinical outcomes. Furthermore, high miR-552 expression predicted poor prognosis in GC patients. Functionally, upregulation of miR-552 promoted cell viability and metastasis in GC. Moreover, miR-552 activated EMT and enhanced p-PI3K and p-AKT expression in GC. In addition, miR-552 overexpression was found to reduce Bax expression and promote survival gene Bcl-2 expression in GC cells. Briefly, miR-552 plays a carcinogenic role in the progression of GC.

Consistent with our results, upregulation of miR-552 had been examined in colorectal cancer and osteosarcoma [10, 22]. In addition, increased expression of miR-552 was associated with a poor prognosis of colorectal cancer [12], which is the same as our results. Functionally, miR-552 was reported to promote the proliferation and EMT of hepatocellular carcinoma cells [23]. Wang et al. demonstrated that miR-552 enhanced the metastatic capacity of colorectal cancer cells [24]. Here, the acceleration of proliferation, migration, and invasion of GC cells as well as EMT was also induced by miR-552. In addition, we also found that miR-552 regulated apoptosis-associated proteins (Bcl-2/Bax) and PI3K/AKT pathway to be involved in GC progression, which has not been reported in previous studies. Besides that, miR-552 was confirmed to target FOXO1 directly and inversely regulated its expression in GC.

FOXO1, as a target gene, is regulated by some miRNAs in human cancers, such as miR-9 and miR-135a [25, 26]. In particular, miR-132 upregulation was found to promote GC cell growth through suppression of FOXO1 translation [27]. In this study, the upregulation of FOXO1 reversed the carcinogenesis induced by miR-552 in GC. Furthermore, miR-552 overexpression suppressed FOXO1 expression in GC. It indicated that upregulation of miR-552 accelerated GC progression by downregulation of FOXO1. Besides that, FOXO1 has been shown to function as a tumor inhibitor in many cancers, including GC [28, 29]. These results also indicated that miR-552 served as a tumor promoter in GC by inhibiting FOXO1 expression. In addition, previous studies showed that miR-96 played an oncogenic role in papillary thyroid carcinoma by regulating AKT/FOXO1 pathway [30]. In our study, miR-552 also accelerated the progression of GC by regulating the FOXO1/PI3K/AKT pathway.

## 5. Conclusion

In summary, this study proposed the upregulation of miR-552 in GC, which was related to poor prognosis in GC patients. Functionally, miR-552 promoted cell viability and metastasis and activated EMT and I3K/AKT in GC via targeting FOXO1. Although we have preliminarily evaluated the regulatory mechanism of miR-552, a more in-depth study of miR-552 in GC is still essential.

## Figures and Tables

**Table 1:** Relationship between miR-552 expression and their clinic-pathological characteristics in GC patients.

Characteristics	Cases	miR-552		*p*-value
		High	Low	
Age (years)				0.08
≥60	38	20	18	
<60	46	30	16	
Gender				0.15
Male	58	32	26	
Female	26	18	8	
Tumor size (mm)				0.21
≤5.0	31	19	12	
>5.0	53	31	22	
Differentiation				0.009*∗*
Well/moderate	24	18	6	
Poor	60	32	28	
Lymph node metastasis				0.04*∗*
Yes	62	42	20	
No	22	8	14	
TNM stage				0.02*∗*
I-II	28	18	10	
III-IV	56	32	24	

Statistical analyses were performed by the *χ*^2^ test. ^*∗*^*p* < 0.05 was considered significant.

## Data Availability

The datasets used and/or analyzed during the current study are available from the corresponding author on reasonable request.

## References

[1] Arnold M., Moore S. P., Hassler S., Ellison-Loschmann L., Forman D., Bray F. (2014). The burden of stomach cancer in indigenous populations: a systematic review and global assessment. *Gut*.

[2] Fock K. M., Ang T. L. (2010). Epidemiology ofHelicobacter pyloriinfection and gastric cancer in Asia. *Journal of Gastroenterology and Hepatology*.

[3] Kim J. G., Ryoo B. Y., Park Y. H. (2008). Prognostic factors for survival of patients with advanced gastric cancer treated with cisplatin-based chemotherapy. *Cancer Chemotherapy and Pharmacology*.

[4] Cervantes A., Roda D., Tarazona N., Roselló S., Pérez-Fidalgo J. A. (2013). Current questions for the treatment of advanced gastric cancer. *Cancer Treatment Reviews*.

[5] Liu H., Lei C., He Q., Pan Z., Xiao D., Tao Y. (2018). Nuclear functions of mammalian MicroRNAs in gene regulation, immunity and cancer. *Molecular Cancer*.

[6] Yang H., Fu H., Wang B. (2018). Exosomal miR-423-5p targets SUFU to promote cancer growth and metastasis and serves as a novel marker for gastric cancer. *Molecular Carcinogenesis*.

[7] An J. X., Ma M. H., Zhang C. D., Shao S., Zhou N. M., Dai D. Q. (2018). miR-1236-3p inhibits invasion and metastasis in gastric cancer by targeting MTA2. *Cancer Cell International*.

[8] Miao L., Yao H., Li C. (2016). A dual inhibition: microRNA-552 suppresses both transcription and translation of cytochrome P450 2E1. *Biochimica et Biophysica Acta (BBA)—Gene Regulatory Mechanisms*.

[9] Kim H. K., Lim N. J., Jang S. G., Lee G. K., Lee G. K. (2014). miR-592 and miR-552 can distinguish between primary lung adenocarcinoma and colorectal cancer metastases in the lung. *Anticancer Research*.

[10] Chao Y., Hu K., Wang X., Wang L. (2019). MicroRNA-552 promotes migration and invasion of osteosarcoma through targeting TIMP2. *Biochemical and Biophysical Research Communications*.

[11] Cai W., Xu Y., Yin J., Zuo W., Su Z. (2019). miR-552-5p facilitates osteosarcoma cell proliferation and metastasis by targeting WIF1. *Experimental and Therapeutic Medicine*.

[12] Wang N., Liu W. (2018). Increased expression of miR-552 acts as a potential predictor biomarker for poor prognosis of colorectal cancer. *European Review for Medical and Pharmacological Sciences*.

[13] Coomans de Brachène A., Demoulin J.-B. (2016). FOXO transcription factors in cancer development and therapy. *Cellular and Molecular Life Sciences*.

[14] Wu Y., Elshimali Y., Sarkissyan M., Mohamed H., Clayton S., Vadgama J. V. (2012). Expression of FOXO1 is associated with GATA3 and Annexin-1 and predicts disease-free survival in breast cancer. *American Journal of Cancer Research*.

[15] Xie L., Ushmorov A., Leithäuser F. (2012). FOXO1 is a tumor suppressor in classical Hodgkin lymphoma. *Blood*.

[16] Zaballos M. A., Santisteban P. (2013). FOXO1 controls thyroid cell proliferation in response to TSH and IGF-I and is involved in thyroid tumorigenesis. *Molecular Endocrinology*.

[17] Guttilla I. K., White B. A. (2009). Coordinate regulation of FOXO1 by miR-27a, miR-96, and miR-182 in breast cancer cells. *Journal of Biological Chemistry*.

[18] Guo Y., Liu H., Zhang H., Shang C., Song Y. (2012). miR-96 regulates FOXO1-mediated cell apoptosis in bladder cancer. *Oncology Letters*.

[19] Burgering B. M. T., Medema R. H. (2003). Decisions on life and death: FOXO Forkhead transcription factors are in command when PKB/Akt is off duty. *Journal of Leukocyte Biology*.

[20] Lian R., Lu B., Jiao L. (2016). MiR-132 plays an oncogenic role in laryngeal squamous cell carcinoma by targeting FOXO1 and activating the PI3K/AKT pathway. *European Journal of Pharmacology*.

[21] Cui H.-B., Ge H.-E., Wang Y.-S., Bai X.-Y. (2018). MiR-208a enhances cell proliferation and invasion of gastric cancer by targeting SFRP1 and negatively regulating MEG3. *The International Journal of Biochemistry & Cell Biology*.

[22] Kwak B., Kim D. U., Kim T. O., Kim H. S., Kim S. W. (2018). MicroRNA-552 links Wnt signaling to p53 tumor suppressor in colorectal cancer. *International Journal of Oncology*.

[23] Qu W., Wen X., Su K., Gou W. (2019). MiR-552 promotes the proliferation, migration and EMT of hepatocellular carcinoma cells by inhibiting AJAP1 expression. *Journal of Cellular and Molecular Medicine*.

[24] Wang J., Li H., Wang Y. (2016). MicroRNA-552 enhances metastatic capacity of colorectal cancer cells by targeting a disintegrin and metalloprotease 28. *Oncotarget*.

[25] Liu D.-Z., Chang B., Li X.-D., Zhang Q.-H., Zou Y.-H. (2017). MicroRNA-9 promotes the proliferation, migration, and invasion of breast cancer cells via down-regulating FOXO1. *Clinical and Translational Oncology*.

[26] Zeng Y. B., Liang X. H., Zhang G. X. (2016). miRNA-135a promotes hepatocellular carcinoma cell migration and invasion by targeting forkhead box O1. *Cancer Cell International*.

[27] Li W., Zhang J., Chen T., Yin P., Yang J., Cao Y. (2015). miR-132 upregulation promotes gastric cancer cell growth through suppression of FoxO1 translation. *Tumor Biology*.

[28] Zang Y., Wang T., Pan J., Gao F. (2017). miR-215 promotes cell migration and invasion of gastric cancer cell lines by targeting FOXO1. *Neoplasma*.

[29] Yang X. W., Shen G. Z., Cao L. Q. (2014). MicroRNA-1269 promotes proliferation in human hepatocellular carcinoma via downregulation of FOXO1. *BMC Cancer*.

[30] Song H. M., Luo Y., Li D. F. (2015). MicroRNA-96 plays an oncogenic role by targeting FOXO1 and regulating AKT/FOXO1/Bim pathway in papillary thyroid carcinoma cells. *International Journal of Clinical and Experimental Pathology*.

